# The expression and relaxant effect of bitter taste receptors in human bronchi

**DOI:** 10.1186/1465-9921-14-134

**Published:** 2013-11-22

**Authors:** Stanislas Grassin-Delyle, Charlotte Abrial, Sarah Fayad-Kobeissi, Marion Brollo, Christophe Faisy, Jean-Claude Alvarez, Emmanuel Naline, Philippe Devillier

**Affiliations:** 1 Laboratoire de Pharmacologie Respiratoire UPRES EA220, Hôpital Foch, 11 rue Guillaume Lenoir, F-92150 Suresnes, Paris, France; 2 Réanimation Médicale, Hôpital Européen Georges Pompidou, 20 rue Leblanc, 75015 Paris, France; 3 Université Sorbonne Paris Cité, Paris, France; 4 Laboratoire de Pharmacologie-Toxicologie, Hôpital Raymond Poincaré, 104 boulevard Raymond Poincaré, 92380 Garches, France; 5 Université Versailles Saint-Quentin, UFR Sciences de la Santé Simone Veil, 2 avenue de la Source de la Bièvre, 78180 Montigny-Le-Bretonneux, France

**Keywords:** Bitter taste receptors, Human bronchi, Relaxation

## Abstract

**Background:**

Bitter-taste receptors (TAS2Rs) have recently been involved in the relaxation of mouse and guinea pig airways, and increased expression of TAS2Rs was shown in blood leucocytes from asthmatic children. We sought to identify and characterize the TAS2Rs expressed in isolated human bronchi and the subtypes involved in relaxation.

**Methods:**

Human bronchi were isolated from resected lungs and TAS2R transcripts were assessed with RT-qPCR. Relaxation to TAS2R agonists was tested in organ bath in the presence or absence of pharmacological modulators of the signalling pathways involved in bronchial relaxation.

**Results:**

We detected the expression of TAS2R transcripts in human bronchi. The non-selective agonists chloroquine, quinine, caffeine, strychnine and diphenidol produced a bronchial relaxation as effective and potent as theophylline but much less potent than formoterol and isoproterenol. Denatonium, saccharin and colchicine did not produce relaxation. Receptor expression analysis together with the use of selective agonists suggest a predominant role for TAS2R5, 10 and 14 in bitter taste agonist-induced relaxation. The mechanism of relaxation was independent of the signalling pathways modulated by conventional bronchodilators and may be partly explained by the inhibition of phosphatidylinositol-3-kinases.

**Conclusions:**

The TAS2Rs may constitute a new therapeutic target in chronic obstructive lung diseases such as asthma.

## Background

Lung diseases such as asthma and chronic obstructive pulmonary disease (COPD) are inflammatory diseases characterized by airway obstruction and airflow limitation. Besides corticosteroids, bronchodilators are thus first-line therapies for their pharmacological management. The current cornerstone of bronchodilators is β_2_-adrenoreceptor agonists, but several issues were raised such as tachyphylaxis or long-term safety. Furthermore, even if β_2_-adrenoreceptor agonists provide short-term relief for airflow limitation, their actions to treat the underlying pathology is limited, if any. The development of novel therapies would thus be desirable, even more with therapies acting on both the inflammatory and obstructive components of the disease. To this end, bitter taste receptors (TAS2Rs) may be a target of interest since, in addition to their recently described bronchodilator and anti-inflammatory properties [1,2], their increased expression was shown in peripheral blood leucocytes of asthmatic children [3]. The TAS2Rs constitute a family of around 25 G-protein coupled receptors (GPCRs) that share between 30% and 70% amino acid sequence homology [4]. The TAS2Rs vary in their selectivity towards bitter compounds: some subtypes are restricted selective to a few molecules, whereas some others respond to a wide range. Correspondingly, some bitter compounds are known to be agonists for a single TAS2R subtype, whereas others activate a substantial number of receptors [5]. More than a hundred molecules (including chloroquine, caffeine, strychnine, colchicine and erythromycin) have been described as TAS2R agonists. The TAS2R19, 41, 42, 45 and 60 subtypes are considered to be orphan receptors, since no cognate agonists have yet been identified. The TAS2R intracellular domain is coupled to gustducin, an heterotrimeric G-protein (consisting of α, β and γ subunits) that is characteristic of taste reception [6]. The α-gustducin subunit may be coupled to phosphodiesterases involved in the regulation of intracellular cyclic nucleotide levels. The β/γ subunits are able to activate phospholipase Cβ2 (PLCβ2), leading to the generation of inositol triphosphate and the release of intracellular calcium [7,8].

The unexpected expression of TAS2Rs in airway epithelium and smooth muscle cells was recently documented [1,9], and bitter taste receptor agonists have been shown to induce a relaxation of pre-contracted mouse airways and guinea pig trachea [1,10]. The relaxation of mouse airways by bitter taste receptor agonists was three-fold greater than that elicited by the β_2−_adrenoreceptor agonist isoproterenol [1]. However, the pharmacological activity of a given TAS2R agonist may differ from one species to another, as illustrated by the example of saccharin (which relaxes mouse airways but not guinea pig airways [1,10]). Studies on isolated human tissues are rare and have generated contradictory findings. Although Deshpande *et al*. confirmed their observations for chloroquine and saccharin on human bronchi [1], Belvisi *et al*. and Morice *et al*. reported that (i) chloroquine-induced relaxation was less potent than that of isoproterenol and (ii) saccharin was devoid of effect [11,12]. Furthermore, attempts to identify the signalling pathways involved in the TAS2Rs-mediated relaxation were relatively unsuccessful. Paradoxically, the stimulation of bitter taste receptors in human airway smooth muscle cells induced relaxation following a localized increase in intracellular calcium, which in turn caused membrane hyperpolarization via the activation of large conductance potassium channels (BK_Ca_) [1]. This observation was then partly confirmed in studies of mouse [13,14] and guinea pig airways [10] while another most recent hypothesis to explain the relaxant effect of chloroquine in mouse airways was the inhibition of L-type voltage-gated calcium channels [15]. Altogether, these data demonstrate that the exact mechanism of bitter taste-induced airway relaxation remains poorly known - particularly in human whole tissues. The objectives of the present study were to (i) characterize TAS2R expression in isolated human bronchi, (ii) describe the relaxant effect and (iii) establish which pathways are involved in TAS2R-mediated bronchial relaxation.

## Materials and methods

### Drugs and chemicals

The TAS2R agonists chloroquine diphosphate, quinine hydrochloride dihydrate, saccharin sodium hydrate, denatonium benzoate, 1,10-phenanthroline hydrochloride monohydrate, caffeine, colchicine, ofloxacin, malvidin-3-glucoside, strychnine hemisulphate, erythromycin, dapsone, carisoprodol, flufenamic acid and sodium cromoglycate were obtained from Sigma-Aldrich (Saint Quentin Fallavier, France) and diphenidol hydrochloride was provided by TCI Europe (Zwijndrecht, Belgium). The control relaxants (formoterol fumarate dihydrate, isoproterenol hydrochloride, theophylline monohydrate) and constrictors (histamine dihydrochloride and acetylcholine chloride) were obtained from Sigma-Aldrich, as were tetraethylammonium chloride, indomethacin and NG-nitro-L-arginine methyl ester hydrochloride (L-NAME). H89 dihydrochloride, U73122 hydrate, iberiotoxin, thapsigargin, BAY K8644, oubain, wortmannin, PI-828, 740 Y-P and brefeldin A were purchased from Tocris (Bristol, UK). All products were solubilized and diluted in sterile water, with the exception of erythromycin, dapsone, carisoprodol, flufenamic acid, thapsigargin, BAY K8644, ouabain, wortmannin and PI-828, which were solubilized in DMSO and then diluted in water. The maximum final concentrations of DMSO in the organ bath had no effect on bronchial contractility.

### Obtainment of human bronchi

Human lung tissue was obtained from macroscopically healthy parts of the lungs from 77 patients (51 men and 26 women; age range: 44–83; mean age: 64 ± 9) undergoing surgical resection for lung carcinoma at Foch Hospital (Suresnes, France) or the Val d'Or Clinic (Saint Cloud, France). The use of resected lung tissues for research purposes was approved by the local institutional review board (*Comité de Protection des Personnes Ile de France VIII*, Boulogne Billancourt, France).

### Reverse transcriptase – quantitative polymerase Chain reaction (RT-qPCR) analysis

RT-qPCR experiments were performed as previously described with some modifications [16]. Bronchial segments were crushed and homogenized in TRIzol^®^ reagent immediately after dissection, using a ball mill TissueLyser LT (Qiagen Courtaboeuf, France). Total RNA was extracted from bronchus homogenates using TRIzol^®^. The amount of RNA extracted was estimated by spectrophotometry at 260 nm (Biowave DNA; Biochrom, Cambridge, England) and its quality was assessed in a microfluidic electrophoresis system (RNA Standard Sensitivity kits for Experion^®^, BioRad, Marnes-la-Coquette, France). After treatment with DNase I (Life Technologies, Saint Aubin, France), 1 μg of total RNA was subjected to reverse transcription (SuperScript^®^ III First-Strand SuperMix kit, Life Technologies). The resulting cDNA was then used for quantitative real-time PCR experiments with TaqMan^®^ chemistry (Life Technologies). The amplification was carried out using 20 ng cDNA (Gene Expression Master Mix, Life Technologies) in a StepOnePlus thermocycler (Life Technologies). The conditions were as follows: initial denaturation at 95°C for 10 min followed by 40 cycles of annealing/extension (95°C for 15 s and then 60°C for 1 min). Fluorescence was measured at each cycle and the threshold cycle (Ct) of the real-time PCR was defined as the point at which a fluorescence signal corresponding to the amplification of a PCR product was detectable. The reaction volume was set at 10 μL. The expression of transcripts of the genes of sixteen TAS2Rs (*TAS2R3*, *TAS2R4*, *TAS2R5*, *TAS2R7*, *TAS2R8*, *TAS2R9*, *TAS2R10*, *TAS2R14*, *TAS2R19*, *TAS2R20*, *TAS2R31*, *TAS2R38*, *TAS2R39*, *TAS2R43 TAS2R45*, and *TAS2R46*) has been analysed in the bronchi using a specific TaqMan^®^ array based on predesigned reagents (Assay-on-Demand^®^, Life Technologies). In order to validate the extraction of intact cellular mRNA and standardize the quantitative data, three reference genes (those for hypoxanthine phosphoribosyltransferase (*HPRT1*), glyceraldehyde-3-phosphate dehydrogenase (*GAPDH*) and β-glucuronidase (*GUSB*)) were amplified as the same time.

### Preparation of tissues for organ bath studies

The bronchi were dissected, cleaned and cut into segments of identical length and diameter, as previously described [17], with a technique which was previously shown to preserve a functional epithelium [18]. Only bronchial segments far from the tumour area and with an inner diameter of between 1 mm and 3 mm were selected. Before use, the segments were stored at +4°C in a Krebs-Henseleit solution (NaCl 119 mM, 5.4 mM KCl, 2.5 mM CaCl_2,_ 1.2 mM KH_2_PO_4_, 1.2 mM MgSO_4,_ 25 mM NaHCO_3_ and 11.7 mM glucose). On the following day, human bronchial segments were placed in isolated organ bath filled with 5 mL of Krebs-Henseleit solution, oxygenated with 95%/5% O_2_/CO_2_ and thermostated at 37°C. Tension was measured isometrically with a strain gauge (UF1; Piodem, Canterbury, Kent, UK) connected to an amplifier (EMKA Technologies France, Paris). Data were acquired, processed and analysed with a computerized system running IOX v1.56.8 and Datanalyst v1.58 software (EMKA Technologies France). An initial load of about 3 g was applied to each segment, which rapidly fell down to a basal tone comprised between 1.5 and 2.5 g during the stabilisation period, when the preparations were allowed to stand for thirty minutes with renewal of the Krebs-Henseleit solution every ten minutes. In a first set of experiments, the bronchi were pre-contracted with 10 μM histamine. Increasing concentrations of bitter taste receptor agonists (chloroquine, quinine, denatonium, colchicine, phenanthroline, strychnine, diphenidol, ofloxacin, caffeine and saccharin) or known bronchial relaxants (isoproterenol, formoterol and theophylline) were then added when the equilibrium tension of the previous concentration was reached (generally every 7–10 minutes). After the last concentration level of bitter taste receptor agonists or relaxants, the maximum relaxation of each segment was evaluated by the addition of 3 mM theophylline. In this set of experiments, each compound was tested on a bronchial ring from each patient.

In a second set of experiments, the signalling pathways involved in the relaxation observed with chloroquine and phenanthroline (see below) were investigated. After an initial equilibration period, bronchi were incubated for 30 min (unless otherwise stated) in the presence of modulators of potassium channels (0.1 μM iberiotoxin, 1 mM tetraethylammonium), calcium signalling (0.1 μM thapsigargin, 1 μM U73122, 1 μM BAY K8644), Na^+^/K^+^ ATPase (10 μM ouabain), protein kinase A (10 μM H89 overnight), exchange proteins directly activated by cAMP (Epacs) (20 μM brefeldin A overnight), phosphoinositide 3-kinases (0.5 μM wortmannin, 2 μM PI-828 overnight), cyclooxygenases (1 μM indomethacin) or nitric oxide synthetase (3 mM NG-nitro-L-arginine methyl ester hydrochloride (L-NAME)) prior to pre-contraction with 10 μM histamine and then the step-wise addition of increasing concentrations of TAS2R agonists. In a third second set of experiments designed to assess the epithelium's role in the relaxation caused by bitter taste receptor agonists, the bronchial epithelium was stripped from bronchial rings from each patient by carefully scraping the luminal surface with a cotton pad soaked in Krebs solution. The relaxation induced by TAS2R agonists was compared with the relaxation of segments from the same bronchi without epithelium stripping. Each of the latter experiments was performed in duplicate.

### Statistical analysis

Values in the text and figures are expressed as the arithmetic mean ± the standard error of the mean (SEM) from experiments with bronchi from *n* independent donors. Changes in muscle tone (E) were expressed as a percentage of the relaxation obtained with 3 mM theophylline. E_max_ corresponds to the E value obtained with the highest agonist concentration tested. The potency (pD_2_) of agonists was defined as the negative logarithm of the molar concentration of agonist producing 50% of the maximal effect (EC_50_) and was calculated from the concentration-response curves. Sigmoidal concentration-response curves were plotted and analysed with GraphPad Prism software (version 5.01, GraphPad Software^®^, San Diego, CA, USA) by non-linear regression.

The quantitative data obtained from RT-qPCR experiments was expressed as relative expression (2^-ΔCt^) [19], where ΔC_t_ is the difference between the target gene C_t_ and the mean C_t_ of the reference genes.

Data were evaluated statistically in an analysis of variance and Dunnett’s post-test. A difference was considered statistically significant when the probability value *p* was below 0.05 (*p* <0.05). In the Figures, the statistical significance of a given comparison is indicated by the symbol * (*p* <0.05), ** (*p* <0.01) or *** (*p* <0.001).

## Results

### Expression of bitter taste receptor gene transcripts in human bronchi

**Figure 1:**
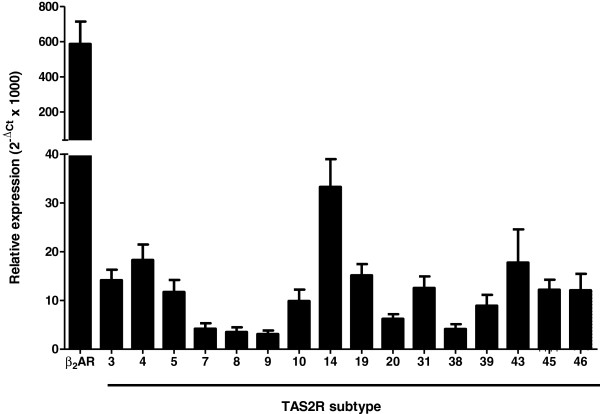
**Relative expression (2**^**-ΔCt **^**× 1000) of β**_**2**_**-adrenoreceptor (β**_**2**_**AR) and *TAS2R3*, *4*, *5*, *7*, *8*, *9*, *10*, *14*, *19*, *20*, *31*, *38*, *39*, *43*, *45 *and *46 *gene transcripts in human bronchi (*n*** **= 9-14).** All transcripts were found expressed in all patients, except *TAS2R9*, *43* and *46* which were found in bronchi from 8/9, 9/14 and 8/9 patients only.

Bronchial expression of the gene transcripts of the β_2_-adrenoreceptor and sixteen TAS2Rs is summarized in Figure 1. Transcripts of genes coding for bitter taste receptors were identified in the bronchi of all patients, except those of *TAS2R9*, *43* and *46* found in bronchi from 8/9, 9/14 and 8/9 patients only. The mRNA of the β_2_-adrenoreceptor was detected in the bronchi of all patients, with a mean relative expression 19-fold greater than the expression of the most abundant TAS2R (*TAS2R14*).

### Effects of bitter taste receptor agonists on the contractility of human bronchi

**Figure 2:**
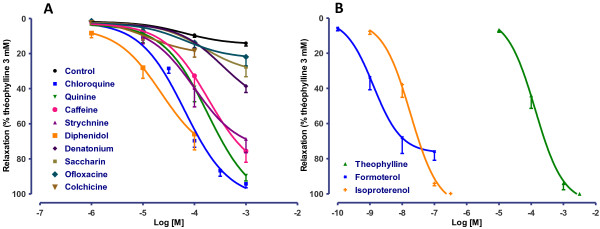
**Concentration-response curves for TAS2R agonists (chloroquine, quinine, caffeine, strychnine, denatonium, saccharin and ofloxacin (all 1 μM to 1 mM) and colchicine and diphenidol (1 μM to 0.1 mM)) on human bronchi (A), and reference relaxants (theophylline from 10 μM to 3 mM, formoterol from 0.1 nM to 0.1 μM and isoproterenol from 1 nM to 0.3 μM) (B).** Control curves correspond to experiments in which only vehicle (water) was added to the organ bath. The results with TAS2R agonists and reference molecules are expressed as a percentage of the relaxation observed with 3 mM theophylline (mean ± SEM from 9 to 24 independent experiments).

In the first set of experiments, we used non-selective TAS2R agonists (chloroquine, quinine, denatonium, colchicine, strychnine, diphenidol, caffeine and saccharin) to cover the widest possible range of receptors (Table 1). Chloroquine, quinine, caffeine, strychnine and diphenidol elicited marked, concentration-dependent relaxation of human bronchi (Figure 2A). The maximum effect was significantly greater than the weak, spontaneous relaxation over time observed with control bronchi. As shown in Table 2, the E_max_ values for TAS2R agonists (between 66% to 94%) were close to those observed with β_2−_adrenoreceptor agonists isoproterenol (99%) and formoterol (76%) and with theophylline (100%, by definition) (Figure 2B). The pD_2_ values of the TAS2R agonists ranged from 4.6 ± 0.4 (diphenidol) and 3.7 ± 0.3 (caffeine and quinine); these were close to that of theophylline (3.9 ± 0.1) but much lower than the pD_2_ values of formoterol and isoproterenol (8.9 ± 0.1 and 7.7 ± 0.1 respectively). In contrast, the E_max_ values for other TAS2R agonists (denatonium, saccharin, ofloxacin and colchicine) did not differ significant from controls. We also investigated the influence of bronchi diameter on the relaxation to bitter agonists. Chloroquine and phenanthroline relax with the same efficacy and potency bronchi with diameter smaller than 1 mm and larger than 5 mm (*n* = 5; not shown).

**Table 1:** Compounds selected for functional studies in view of their agonistic properties for the 25 TAS2R receptors identified in humans to date

***hTAS2R***	**1**	**3**	**4**	**5**	**7**	**8**	**9**	**10**	**13**	**14**	**16**	**19**	**20**	**30**	**31**	**38**	**39**	**40**	**41**	**42**	**43**	**45**	**46**	**50**	**60**
**Chloroquine**		10 and 172 ± 29			+			10000									100								
**Quinine**			10		10			10		10					10		10	10			10		10		
**Denatonium**			300			1000		3 and 120 ± 56	30					0.03 and 0.27 ± 0.06			100				300		30 and 240 ± 192		
**Colchicine**			100 and 1025 ± 121														3000						300 and 1580 ± 170		
**Strychnine**					+			3 and 21.8 ± 7.5															0.1 and 0.43 ± 0.02		
**Diphenidol**	100		100		10			30	30	10	100		100	100	3	100	100	30			30		30		
**Caffeine**					300			300		300											300		300		
**Saccharin**						+									170 ± 10						80 ± 60				
**Ofloxacin**							EC_50_ ≈200																		
**Phenanthroline**				100																					
**Erythromycin**								300																	
**Dapsone**			100					100										30							
**Flufenamic acid**										0.01 and 0.14 ± 0.02															
**Carisoprodol**										100															
**Malvidin-3-glucoside**					6 and 12.6 ± 0.7																				
**Sodium cromoglycate**					3000 and 4500 ± 1600								10 and 45 ± 25								3000				

The most recent nomenclature was adopted [5,20-24]. Unless otherwise stated, single values (without SEM) represent the threshold concentration, defined as the lowest concentration (μM) that elicited a calcium response in transfected HEK-293 T cells, whereas values presented as mean ± SEM represent the EC_50_ (μM) [5]. When both threshold concentration and EC_50_ were available, both values were reported in this order. When no quantitative indicator was available, the symbol “+” indicates agonist property of the compound to the receptor.

**Table 2:** **Maximum relaxation (E**_**max**_**) and potency (pD**_**2**_**) observed with non-selective TAS2R agonists and reference relaxing agents (isoproterenol, formoterol and theophylline) on human bronchi**

	**E**_**max **_**(%)**	**pD**_**2**_	***n***
**Control**	15.0 ± 3.5	NA	11
**Chloroquine**	94.2 ± 2.3	4.2 ± 0.1	10-24
**Quinine**	89.5 ± 3.0	3.7 ± 0.2	9
**Caffeine**	75.6 ± 6.3	3.7 ± 0.3	11
**Strychnine**	68.7 ± 7.8	4.0 ± 0.4	10
**Diphenidol**	65.9 ± 9.0	4.6 ± 0.4	11
**Denatonium**	38.6 ± 3.6	NA	11
**Saccharin**	27.6 ± 5.5	NA	10
**Ofloxacin**	21.9 ± 5.1	NA	11
**Colchicine**	18.3 ± 3.7	NA	10
**Isoproterenol**	99.8 ± 0.2	7.7 ± 0.1	11
**Formoterol**	75.9 ± 5.0	8.9 ± 0.2	11
**Theophylline**	100.0 ± 0.0	3.9 ± 0.1	10

E_max_ values are expressed as a percentage of the relaxation observed with 3 mM theophylline (mean ± SEM of experiments performed on isolated bronchi from *n* distinct patients).

### Characterization of receptor subtypes involved in the relaxant response

**Figure 3:**
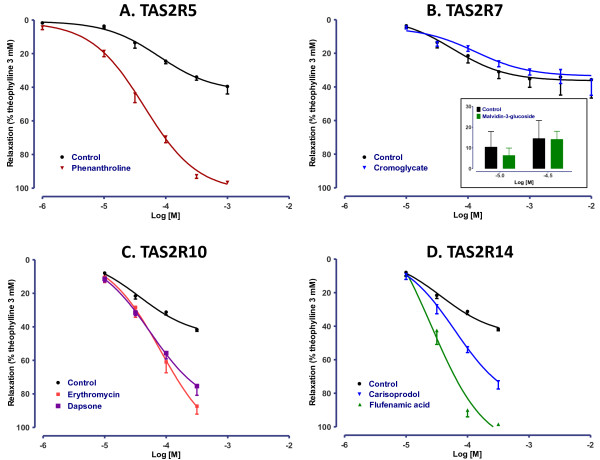
**Concentration-response curves of agonists selective for (A) TAS2R5 (phenanthroline, 10 μM to 1 mM), (B) TAS2R7 (sodium cromoglycate, 10 μM to 10 mM and malvidin-3-glucoside, 10 and 30 μM), (C) TAS2R10 (erythromycin and dapsone, 10 μM to 0.5 mM) and (D) TAS2R14 (carisoprodol and flufenamic acid, 10 μM to 0.5 mM).** Control curves correspond to experiments in which only vehicle (DMSO or water) was added to the organ bath. The results are expressed as a percentage of the relaxation observed with 3 mM theophylline (mean ± SEM from 3 to 22 independent experiments).

The receptor expression results and the above-mentioned effects of certain TAS2R agonists suggested the involvement of TAS2R7, 10 and 14 in the relaxation of human bronchi. This hypothesis was further investigated with the use of relatively selective agonists. The involvement of TAS2R5 was also probed with phenanthroline; in addition to being selective for this receptor, phenanthroline is the only TAS2R5 agonist to have been described to date [5]. The selective agonists of TAS2R5 (phenanthroline), TAS2R10 (erythromycin and dapsone) and TAS2R14 (carisoprodol and flufenamic acid) induced the relaxation of human bronchi, whereas the TAS2R7 agonists cromoglycate and malvidin-3-glucoside were ineffective up to 10 mM and 30 μM respectively (Figure 3). The potency was similar for the TAS2R5, -10 and −14 agonists, with pD_2_ values of 4.3 ± 0.1, 4.2 ± 0.1 and 4.7 ± 0.2 for phenanthroline, erythromycin and flufenamic acid, respectively (Figure 3).

### Reversibility of the relaxation

**Figure 4:**
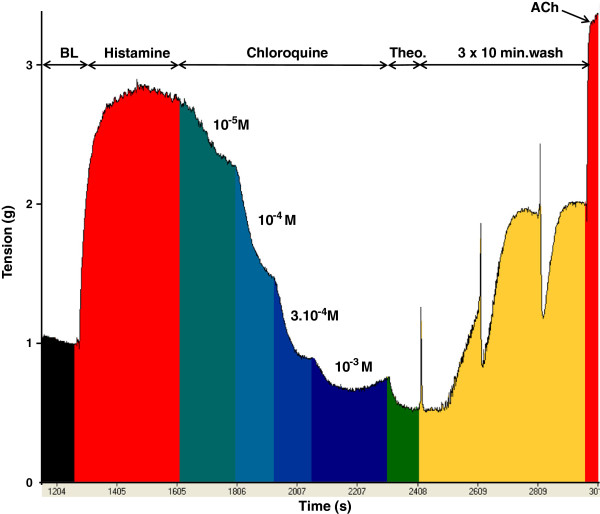
**Representative relaxant profile obtained after application of increasing concentrations of the bitter taste receptor agonist chloroquine in histamine-pre-contracted human bronchi.** Importantly, introduction of 3 mM acetylcholine (ACh) into organ bath at the end of the experiment showed that bronchial function was still present after exposure to TAS2R agonists and a wash-out step. BL: baseline.

When bronchial segments were washed three times with Krebs-Henseleit solution after exposure to the highest concentration of a TAS2R agonist, the tension reverted to its baseline value. Moreover, when 3 mM acetylcholine was applied to the preparations immediately after the wash, a contractile response greater than that obtained with 10 μM histamine was observed and was close to maximum contraction obtained with 3 mM acetylcholine in control experiments (Figure 4). Recovery of baseline tone and contractibility with acetylcholine were observed after exposure to all the TAS2R agonists tested in this study.

### Study of signalling pathways

Since previous experiments had suggested that the relaxation induced by TAS2R agonists was due to opening of BK_Ca_ after activation of the PLCβ pathway and a localized increase in intracellular calcium [1], we investigated the effects of 0.1 μM iberiotoxin (a selective BK_Ca_ inhibitor), 0.1 μM thapsigargin (a sarcoplasmic reticulum Ca^2+^-ATPase inhibitor), 1 mM tetraethylammonium (a non-selective potassium channel inhibitor) and 1 μM U73122 (a PLCβ inhibitor) on the relaxation induced by the bitter taste receptor agonists chloroquine and phenanthroline. None of the inhibitors altered the observed relaxations (Table 3).

**Table 3:** **Maximum relaxation (E**_**max**_**) of human bronchi and potency (pD**_**2**_**) observed with chloroquine or phenanthroline in the presence or absence (control) of U73122 (1 μM, an inhibitor of PLCβ), iberiotoxin (0.1 μM, a selective inhibitor of BK**_**Ca **_**channels), thapsigargin (0.1 μM, an inhibitor of the sarcoplasmic reticulum Ca**^**2+**^**-ATPase), tetraethylammonium (1 mM, a non-selective potassium channels blocker), H89 (10 μM overnight, a PKA inhibitor), brefeldin A (20 μM overnight, an Epac inhibitor), indomethacin (1 µM, a cyclooxygenases inhibitor) and L-NAME (3 mM, a nitric oxide synthetase inhibitor)**

	**Chloroquine**		**Phenanthroline**		
	**E**_**max **_**(%)**	**pD**_**2**_	**E**_**max **_**(%)**	**pD**_**2**_	***n***
**Control**	93 ± 4	4.3 ± 0.2	96 ± 1	4.5 ± 0.1	6-10
**U73122**	96 ± 1	4.3 ±0.2	96 ± 1	4.5 ± 0.1	6
**Iberiotoxin**	96 ± 3	4.2 ± 0.2	97 ± 0.1	4.3 ± 0.2	6
**Thapsigargin**	99 ± 2	4.0 ± 0.1	94 ± 2	4.0 ± 0.2	6
**Tetraethylammonium**	91 ± 3	4.2 ± 0.2	97 ± 1	4.5 ± 0.1	5-6
**H89**	98 ± 1	4.6 ± 0.1	99 ± 1	5.3 ± 0.5	5-6
**Brefeldin A**	92 ± 2	4.6 ± 0.3	97 ± 1	4.5 ± 0.1	5-6
**Indomethacin**	95 ± 2	4.3 ± 0.1	97 ± 1	4.2 ± 0.2	6-7
**L-NAME**	94 ± 3	4.6 ± 0.2	98 ± 1	4.4 ± 0.1	5

E_max_ values are expressed as a percentage of the relaxation observed with 3 mM theophylline (mean ± SEM of experiments performed on isolated bronchi from 5 to 10 patients).

We then focused on other signalling pathways involved in cAMP-dependent human bronchus relaxation. Adenylyl cyclase activation triggers bronchial smooth muscle relaxation following the stimulation of β_2_-adrenergic receptors; it has been reported that TAS2R agonists inhibit the phosphodiesterases responsible for cyclic nucleotide degradation [25]. The downstream effectors activated via a cAMP-dependent mechanism include protein kinase A (PKA), the recently described Epacs and potassium channels (such as BK_Ca_) [26,27]. However, our overnight incubation of human bronchi with the PKA inhibitor H89 (10 μM) or with the Epac inhibitor brefeldin A (20 μM) [28] did not inhibit chloroquine- and phenanthroline-induced relaxation (Table 3). In contrast, the isoproterenol concentration-effect curves were right-shifted by about 0.8 log units with H89 (data not shown).

**Figure 5:**
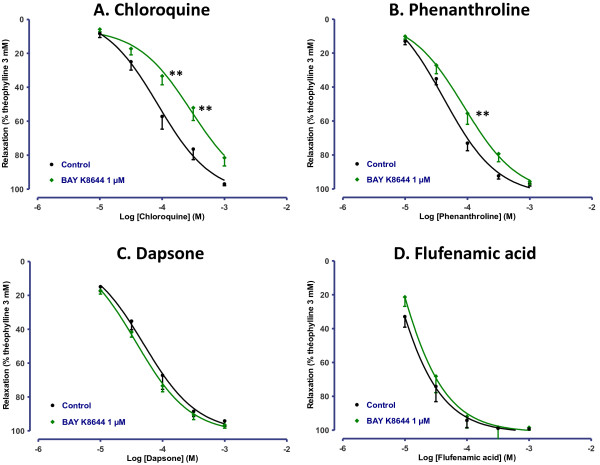
**Concentration-response curves of (A) chloroquine, (B) phenanthroline, (C) dapsone and (D) flufenamic acid in human bronchi pretreated or not with 1 μM BAY K8644.** The results are expressed as a percentage of the relaxation observed with 3 mM theophylline (mean ± SEM from 4 to 10 independent experiments). **p < 0.01.

**Figure 6:**
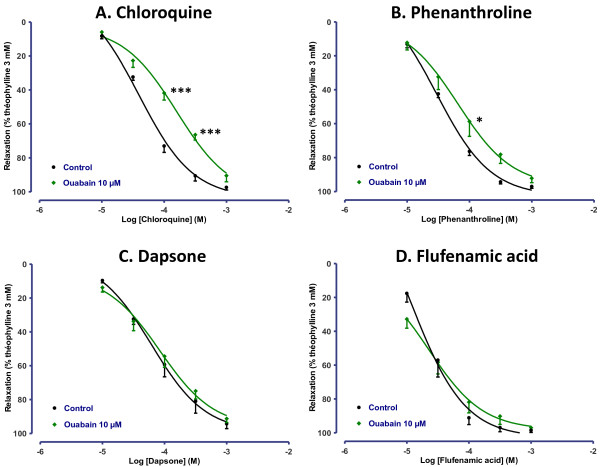
**Concentration-response curves of (A) chloroquine, (B) phenanthroline, (C) dapsone and (D) flufenamic acid in human bronchi pretreated or not with 10 μM ouabain.** The results are expressed as a percentage of the relaxation observed with 3 mM theophylline (mean ± SEM from 5 independent experiments). *p < 0.05; ***p < 0.001.

Recent findings suggested that the relaxation induced by chloroquine in mouse airways may be related to blockade of L-type voltage-gated calcium channels [15]. We thus explored the effects of 1 μM BAY K8644, an activator of L-type voltage-gated calcium channels (Figure 5) as well as those of 10 μM ouabain, an inhibitor of Na^+^/K^+^ ATPAse (Figure 6), which both induce calcium entry in the cell [15,29]. Response profiles were similar with both drugs, which induced a right-shift of concentration-response curves to chloroquine (0.6 and 0.5 log-units respectively) and phenanthroline (0.4 and 0.3 log-units respectively), whereas the response to dapsone and flufenamic acid was unaffected.

**Figure 7:**
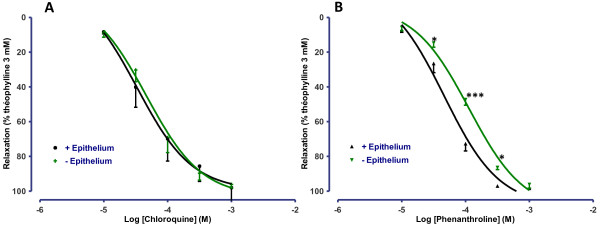
**Concentration-response curves of (A) chloroquine and (B) phenanthroline in human bronchi with or without epithelium.** The results are expressed as a percentage of the relaxation observed with 3 mM theophylline (mean ± SEM from 5 independent experiments). *p < 0.05; ***p < 0.001.

We then explored the involvement of the epithelium and epithelium-dependent signalling pathways, with a focus on prostanoids and nitric oxide. Removal of the bronchial epithelium had no impact on the concentration-response curve for chloroquine (*n* = 5). In contrast, the concentration-response curve for phenanthroline was right-shifted in the absence of epithelium, resulting in a lower pD_2_ (3.9 ± 0.1 in the absence of epithelium *vs*. 4.3 ± 0.1 in the presence of epithelium, *n* = 5; *p* < 0.001) (Figure 7). Pre-incubation of the bronchi with 3 mM L-NAME or 1 μM indomethacin did not significantly alter the response to chloroquine or phenanthroline (Table 3).

**Figure 8:**
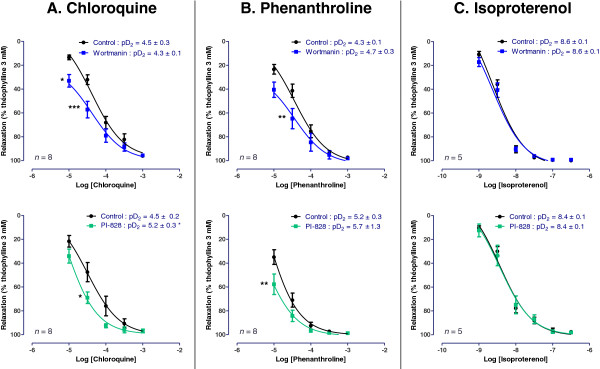
**Concentration-response curves of (A) chloroquine, (B) phenanthroline, and (C) isoproterenol in human bronchi pretreated or not with the PI3K inhibitors wortmannin (0.5 μM) and PI-828 (2 μM overnight).** The results are expressed as a percentage of the relaxation observed with 3 mM theophylline (mean ± SEM from 5 to 8 independent experiments). *p < 0.05; **p < 0.01 and ***p < 0.001 *vs*. control.

We lastly investigated the role of phosphoinositide 3-kinases (PI3Ks), which were previously shown to regulate calcium flux in airway smooth muscle cells [30] and to be involved in the IL-13-induced increase in tracheal contractility in mouse [31]. Wortmanin (0.5 μM) and PI-828 (2 μM overnight) potentiated the relaxation to chloroquine and phenanthroline (Figure 8), which translated into a significant increase in pD_2_ for relaxation to chloroquine in bronchi treated with PI-828 only (4.5 ± 0.2 in the absence of PI-828 *vs*. 5.2 ± 0.3 with PI-828, *n* = 8; *p* < 0.05) (Figure 8). On the other hand, the relaxation to isoproterenol was unaffected by either wortmanin or PI-828.

## Discussion and conclusions

We first demonstrated that (i) TAS2Rs are indeed expressed in human isolated bronchi and (ii) TAS2R agonists trigger relaxation in pre-contracted bronchi. Expression of several TAS2Rs (but not TAS2R7, 38, 39 and 43) has previously been observed in human airway smooth muscle cells [1]. In agreement with the latter findings, we found that not only TAS2R3, 4, 5, 8, 9, 10, 14, 19, 20, 31, 45 and 46 but also TAS2R7, 38, 39 and 43 were expressed in intact bronchi. This result suggests that these four latter subtypes could be expressed by cells other than smooth muscle cells in human bronchi, as has already been observed in epithelial cells [9,32].

A number of TAS2R agonists were found to have relaxant properties in mouse airways and guinea pig trachea [1,10]. Furthermore, chloroquine and saccharin acted as relaxants in bronchial rings from three patients [1], although the latter compound was found to be inactive in another study [11]. We further investigated TAS2R-mediated relaxation in human bronchi by first confirming the relaxation of bronchi exposed to chloroquine. In the present study, quinine, caffeine, strychnine and diphenidol were effective as relaxing agents, whereas saccharin, denatonium, colchicine and ofloxacin were devoid of effect. The tissue relaxation induced by bitter taste compounds was likely to be receptor-mediated effect rather than a non-specific toxic effect because washing the preparations after application of the highest concentration of the TAS2R agonists resulted in the recovery of basal tone and essentially pre-exposure levels of contractility to acetylcholine. Given the current lack of TAS2R antagonists (whether selective for a given subtype or not), we sought to determine which receptor subtypes were primarily involved in the relaxation by combining a receptor gene expression analysis with subtype-selective agonist experiments. In their extensive work with HEK cells transfected with plasmids harboring sequences coding for the different hTAS2R and stably expressing a chimeric G protein subunit (Gα16gust44), Meyerhof *et al*. described the molecular receptive ranges of the 25 human TAS2R with 104 natural or synthetic bitter compounds [5]. Calcium imaging analysis was used as a detection method and quantitative values in this particular model of HEK cells were most often reported as the “threshold concentration”, defined as the minimal concentration that elicited responses from cells but only in rare exceptions were the results expressed as potency (EC_50_ of an agonist for a given receptor). This work was used as a basis for the choice of the different non-selective or subtype-selective agonists used in the present study for which threshold concentration or EC_50_ when available were detailed in Table 1. These data obtained in a transfected renal cell line should only be cautiously extrapolated to experiments performed on human bronchial preparations. For example, many bitter compounds generated artificial calcium responses in HEK cells in the absence of transfected hTAS2R (which prevented from calculating EC_50_ values), and signalling pathways other than changes in intracellular calcium may be activated [5]. Furthermore, the threshold concentrations assessed in HEK cells cannot be easily extrapolated to pharmacological potency (EC_50_ or pD_2_). For example, the threshold concentration of denatorium and strychnine to activate TAS2R10 is 3 μM while the corresponding EC_50_ are 120 ± 56 μM and 21.8 ± 7.5 μM respectively, i.e. a more than 5-fold difference.

Most of the agonists used in the present study activated TAS2R4, 7, 10, 14, 39, 43 and 46 with threshold concentrations in HEK cells mostly between 3 and 300 μM [5], but none was selective for a single receptor subtype. The involvement of TAS2R4, 13, 39, 43 and 46 in bronchial relaxation seems rather unlikely, since concentrations of up to 1 mM denatonium (an agonist of TAS2R4, 13, 39, 43 and 46 with threshold concentrations in HEK cells between 30 and 300 μM for these receptors [5]) and colchicine (an agonist of TAS2R4, 39 and 46 with threshold concentrations between 100 and 3000 μM in HEK cells) were devoid of effect. In human bronchi, the most potent non-selective agonists were chloroquine and diphenidol, followed by quinine, strychnine and caffeine. Phenanthroline (the only known selective agonist of TAS2R5 with threshold concentration of 100 μM) [5] induced relaxation for concentrations as low as 10 μM (10-fold lower than the threshold concentration in HEK cells) suggesting the involvement of TAS2R5. Phenanthroline was at least as effective and potent as chloroquine to relax human bronchi. The TAS2R14 agonists, carisoprodol (also an agonist of TAS2R46) and flufenamic acid, as well as the TAS2R10 agonists erythromycin and dapsone (also an agonist of TAS2R4 and 39) caused equipotent, similarly effective relaxations. A role for TAS2R10 has been previously suggested in ASM by blockade of the strychnine-induced calcium mobilisation by a TAS2R10-raised antibody [1]. In contrast, the involvement of TAS2R7 is unlikely since sodium cromoglycate and malvidin-3-glucoside did not affect bronchial tone for concentrations equivalent or greater than their EC_50_ in HEK cells. A role for TAS2R8, 9 and 31 is also unlikely because of the inactivity of ofloxacin (an agonist of TAS2R9 [20]) and saccharin (TAS2R8, 31 and 43), in agreement with the low expression of these subtype’s transcripts in human bronchi. Similarly, the involvement of receptors TAS2R19, 41, 42, 45 and 60 in the relaxation of human bronchi is unlikely since they are considered orphan receptors and none of the agonists of the present study is known to activate these receptor subtypes [5]. Given the absence of selective agonists for TAS2R1, 3 and 13, the involvement of these latter receptors could not be specifically investigated and thus cannot be formally ruled out.

One limitation of our study relates to the incomplete pharmacological characterization of the available TAS2R agonists. For example, it has also been suggested that chloroquine inhibits airway smooth muscle contractility by inhibiting phospholipase A2 [33]. Caffeine was found to relax airway smooth muscle by direct actin depolymerisation [34] and quinine reportedly bypasses taste receptors and directly activates G-proteins [35]. Likewise, the non-steroidal anti-inflammatory flufenamic acid inhibits the cyclooxygenases responsible for producing prostaglandins (PGEs), which are prominent mediators of bronchial tone. However, flufenamic acid's agonistic properties towards TAS2R14 have been well characterized [5]. Indomethacin, another potent cyclooxygenase inhibitor, was a much less potent relaxant in our model (data not shown). Taken as a whole, these findings suggest that a battery of selective TAS2R agonists and antagonists will be required to confirm our findings and fully elucidate the subtypes of receptors involved in the relaxant response of human bronchi. Our results nevertheless suggest that the TAS2R5, 10 and 14 subtypes may have a prime role in the *in vitro* relaxation of human bronchi, which would be in agreement with the known ability of the TAS2R10 and 14 subtypes to recognise the widest range of bitter compounds [5] and the high transcript expression level of *TAS2R14*.

With respect to drug potency, all the active bitter taste receptor agonists were essentially as potent as theophylline but were much less potent than the β_2_-adrenoreceptors agonists isoproterenol and formoterol (as illustrated by the 3- to 5-log unit difference in pD_2_). These values are in agreement with observations of chloroquine and isoproterenol in human bronchi [12]. However, the agonists were highly effective (relative to theophylline), with E_max_ values greater than 90%; this demonstrates that even though higher concentrations are needed (relative to β_2_-adrenoreceptor agonists), a similar degree of bronchial relaxation can be achieved. Given that the exact mechanism of action of theophylline is still debated and that this compound is known to taste bitter, it cannot be ruled out that TAS2R signalling may also participate in its relaxing activity.

The different pharmacological inhibitors used in the mechanistic part of the study might have impacted precontraction to histamine, and therefore the subsequent relaxation to TAS2R agonists. To analyse the potential relationship between the level of precontraction and the relaxation, we have studied the relaxations to chloroquine as a function of the precontractions induced by 10 μM histamine. On 59 bronchial segments, the relaxation was found independent of the precontraction level (not shown). Hence, the effect of the pharmacological inhibitors on the relaxation to TAS2R agonists (expressed as percentage of maximal relaxation to theophylline) is not related to an indirect effect in link with a potential alteration of the precontraction induced by histamine.

Desphande *et al*. have suggested that relaxation was due to membrane hyperpolarization following the opening of calcium-dependent potassium channels of large conductance and a localized increase in intracellular calcium [1]. The inhibitors of BK_Ca_ channels, sarcoplasmic reticulum Ca^2+^-ATPases and PLCβ used in the present work did not affect chloroquine- or phenanthroline-induced relaxation. Contrasting with findings in smooth muscle cells, these observations suggest that BK_Ca_ signalling is not involved in the TAS2R relaxant response in isolated human bronchi and agree with recent data from experiments on murine airways [14]. However, others modulators of calcium signalling such as ouabain or BAY K8644 revealed differential modulation of TAS2R agonists-induced relaxation, with a clear reduction of chloroquine potency, a more modest reduction of phenanthroline potency, while response to dapsone and flufenamic acid was unaffected. Hence, effects on relaxation to chloroquine may be explained by dependency on the Na^+^/K^+^ exchanger (blocked by ouabain) or on L-type voltage-gated calcium channels, or by a functional antagonism, as a consequence of a rise in intracellular calcium due to the inhibition of the Na^+^/K^+^ exchanger [36] or to the activation of L-type voltage-gated calcium channels. However, since phenanthrolin-induced relaxation was less affected and since dapsone- or flufenamic acid-induced relaxation were not affected at all, non-TAS2R-mediated mechanisms such as effect on potassium, sodium or calcium ion channels [37] or membrane-stabilizing properties [38] may explain the results with chloroquine. These results nevertheless suggest that the described modulation of L-type voltage gated calcium channels [15] is not sufficient to fully explain the TAS2R-induced bronchial relaxation.

The cAMP pathway is obviously a major intracellular signalling pathway in the regulation of bronchial smooth muscle tone. It has been reported that some TAS2R subtypes impair the activity of phosphodiesterases via the gustducin α-subunit. Furthermore, TAS2R receptors may be coupled directly to adenylate cyclase (the enzyme responsible for cAMP synthesis) [6,21,39]. The results of our experiments with pharmacological inhibitors of the cAMP downstream signalling proteins PKA and Epac suggest that these cAMP-dependent pathways are not involved in the TAS2R agonist-related relaxation, which is in agreement with the absence of any increase in the cAMP concentration following the treatment of guinea pig tracheas with TAS2R agonists [10]. Furthermore, endogenous bronchodilators of epithelial origin (such as nitric oxide and PGE_2_) are unlikely to be involved in TAS2R agonist-related relaxation, due to the non-significant effect of nitric oxide synthase and cyclooxygenase inhibitors. In guinea-pig trachea, chloroquine-induced relaxation was also not affected by indomethacin [10]. In our experiments, epithelium removal affected phenanthroline- induced relaxation but not chloroquine-induced relaxation (as already reported for chloroquine in guinea-pig trachea [10]). The relaxation in response to phenanthroline is therefore dependent (at least in part) on an intact epithelium. Phenanthroline is an exclusive TAS2R5 agonist, whereas chloroquine activates a wider range of receptors; hence, receptor expression differences between epithelial cells and smooth muscle cells may explain this result.

We lastly focused on the role of phosphoinositide 3-kinases. The inhibitors of PI3K wortmannin and PI-828 potentiated the relaxation to chloroquine and phenanthroline but did not affect the relaxation to isoproterenol. Wortmannin is described be a non-selective PI3K inhibitor since it also inhibits polo-like kinase family with an IC_50_ in the same range as for PI3K, or other enzymes such as mTOR, myosin light chain kinase (MLCK) and mitogen-activated protein kinase (MAPK); whereas PI-828 selectively targets PI3K. Our data suggest an increase in sensitivity of human bronchi to bitter agonists after incubation with the PI3K inhibitors whereas PI3K do not seem to be involved in the response to β_2_-adrenoreceptor agonists. However, our attempts to induce a right-shift in the concentration-response curves to bitter agonists with the selective PI3K activator 740 Y-P were unsuccessful. This may be explained by both the peptidic nature of the compound (preventing good cell membrane crossing) and to its distinct pharmacological target (it binds to the p85 subunit of PI3K (regulatory factor) whereas wortmannin and PI-828 binds to the p110 subunit (catalytic activity)).

In conclusion, we demonstrated TAS2R transcript expression in human bronchi and identified TAS2R5, 10 and 14 as the subtypes that may be primarily involved in the relaxation of this tissue. Our investigations then showed that none of the signalling pathways targeted by current bronchodilators as well as the inhibition of BK_Ca_ or L-type voltage gated calcium channels could fully explain the TAS2R agonists-induced relaxation of human isolated bronchi. Our observations with PI3K inhibitors suggest that these latter enzymes may be involved in the relaxation to bitter agonists, which would be worth being confirmed with non-peptidic and p110 subunit-selective PI3K’s activators (not available to our knowledge).

The importance of taste signalling in asthma was recently suggested in an analysis of TAS2R expression in peripheral blood leukocytes from asthmatic children [3]. Furthermore, the potential value of TAS2R as a drug target is enhanced by the fact that (at least *in vitro*) TAS2R agonists were effective in relaxing airway smooth muscle even when β_2_-adrenergic receptors (the current cornerstone target of bronchodilators) were subject to tachyphylaxis [40]. The development of selective TAS2R antagonists and more potent, selective TAS2R agonists is nevertheless a prerequisite for better characterizing the TAS2Rs' involvement in relaxation and understanding the corresponding molecular signalling pathways. The many bitter synthetic compounds developed to date [5] may have therapeutic value in obstructive pulmonary diseases through the inhaled route.

## Competing interests

The authors declare that they have no competing interests.

## Authors’ contributions

SGD conceived the study, performed organ bath experiments, analyzed the data, performed the statistical analysis and drafted the manuscript. CA carried out the molecular genetic studies and helped to draft the manuscript. SFK performed organ bath experiments with non-selective agonists, carried out molecular genetic studies and analyzed the data. MB performed molecular genetic studies and analyzed the data. CF analyzed the data and critically revised the manuscript. JCA analyzed the data and critically revised the manuscript. EN performed organ bath experiments, analyzed the data and critically revised the manuscript. PD analyzed the data, critically revised the manuscript and drafted the manuscript. All authors read and approved the final manuscript.
